# 3D Printed Poly(ε-Caprolactone)/Meniscus Extracellular Matrix Composite Scaffold Functionalized With Kartogenin-Releasing PLGA Microspheres for Meniscus Tissue Engineering

**DOI:** 10.3389/fbioe.2021.662381

**Published:** 2021-04-30

**Authors:** Hao Li, Zhiyao Liao, Zhen Yang, Cangjian Gao, Liwei Fu, Pinxue Li, Tianyuan Zhao, Fuyang Cao, Wei Chen, Zhiguo Yuan, Xiang Sui, Shuyun Liu, Quanyi Guo

**Affiliations:** ^1^Institute of Orthopedics, The First Medical Center, Chinese PLA General Hospital, Beijing Key Lab of Regenerative Medicine in Orthopedics, Key Laboratory of Musculoskeletal Trauma and War Injuries PLA, Beijing, China; ^2^School of Medicine, Nankai University, Tianjin, China; ^3^Department of Orthopedics, The First Affiliated Hospital of Zhengzhou University, Zhengzhou, China; ^4^Department of Bone and Joint Surgery, Renji Hospital, School of Medicine, Shanghai Jiao Tong University, Shanghai, China

**Keywords:** PCL, meniscus extracellular matrix, kartogenin, 3D printing, meniscus tissue engineering

## Abstract

Meniscus tissue engineering (MTE) aims to fabricate ideal scaffolds to stimulate the microenvironment for recreating the damaged meniscal tissue. Indeed, favorable mechanical properties, suitable biocompatibility, and inherent chondrogenic capability are crucial in MTE. In this study, we present a composite scaffold by 3D printing a poly(ε-caprolactone) (PCL) scaffold as backbone, followed by injection with the meniscus extracellular matrix (MECM), and modification with kartogenin (KGN)-loaded poly(lactic-co-glycolic) acid (PLGA) microsphere (μS), which serves as a drug delivery system. Therefore, we propose a plan to improve meniscus regeneration via KGN released from the 3D porous PCL/MECM scaffold. The final results showed that the hydrophilicity and bioactivity of the resulting PCL/MECM scaffold were remarkably enhanced. *In vitro* synovium-derived mesenchymal stem cells (SMSCs) experiments suggested that introducing MECM components helped cell adhesion and proliferation and maintained promising ability to induce cell migration. Moreover, KGN-incorporating PLGA microspheres, which were loaded on scaffolds, showed a prolonged release profile and improved the chondrogenic differentiation of SMSCs during the 14-day culture. Particularly, the PCL/MECM-KGN μS seeded by SMSCs showed the highest secretion of total collagen and aggrecan. More importantly, the synergistic effect of the MECM and sustained release of KGN can endow the PCL/MECM-KGN μS scaffolds with not only excellent cell affinity and cell vitality preservation but also chondrogenic activity. Thus, the PCL/MECM-KGN μS scaffolds are expected to have good application prospects in the field of MTE.

## Introduction

The meniscus plays a crucial role in protecting articular cartilage and maintaining joint congruence (Fox et al., 2012, 2015). However, the meniscus damage caused by sports injuries, trauma, and aging may eventually lead to articular cartilage loss and symptomatic osteoarthritis (OA) (Englund et al., 2012; Bilgen et al., 2018). Surgical treatments involving arthroscopic meniscectomy and meniscal allograft transplantation (MAT) are the primary approaches in clinical practice but still present drawbacks (Li H. et al., 2020). Recently, tissue-engineered scaffolding strategies have become one of the mainstream choices in meniscus therapeutic field (Kwon et al., 2019). To date, a wide range of biomaterials, such as synthetic polymers, natural polymers, and tissue-derived materials, have been utilized to fabricate tissue-engineered meniscal scaffold (Makris et al., 2011). Nevertheless, it is well known that meniscus is an anisotropic complex composed of zonal cell phenotypes and extracellular matrix (ECM), which make it hard to mimic microenvironment, hierarchical structure, and morphology of native meniscus (Lee et al., 2014; Zhang et al., 2019). Hence, the construction of a structurally and functionally optimized scaffold with biomimetic microenvironmental characteristics of native meniscus is of great significance for meniscus regeneration.

As a scaffold for MTE, the suitable porous structure is urgently important with regard to the necessity of cell infiltration and growth (Zhang et al., 2016). 3D printing is a promising technology that is capable of individually and accurately manufacturing complex structures (Zhu et al., 2020). Fuse deposition modeling (FDM) has been widely used among these technologies owing to its convenient operation and favorable accuracy (Ngo et al., 2018). Considering the suitable polymers for FDM, poly (ε-caprolactone) (PCL), a kind of biodegradable aliphatic polyester with excellent biocompatibility and mechanical properties, has been utilized as the choice in melt-based extrusion printing systems owing to its proper melting temperature (60°C) and rheological and viscoelastic properties (Visser et al., 2013; Malikmammadov et al., 2018). However, the limited resolution of 3D printed scaffold could not construct an ECM-mimicking porous structure alone. Furthermore, poor hydrophilicity and lack of biochemical cues, as well as avoiding abrasion of the articular cartilage, make the PCL scaffolds require the addition of complementary materials to enhance the biological functions and reduce the frictional force between the scaffold and cartilage to a certain extent. Therefore, the combination of additional materials to form macro–microstructure with improved biological functions has been utilized as an important strategy in tissue engineering (Giannitelli et al., 2015; Zhu et al., 2020).

According to the literature, decellularized extracellular matrix-combined scaffold exhibited more excellent cell adhesion and marked differentiation potential, which was due to its ability to recapitulate most features of natural ECM and regulate cell fate (Cunniffe et al., 2019; Hussey et al., 2018). In addition, decellularized extracellular matrix-only scaffolds possess dissatisfactory mechanical strength, which limits their application in MTE (Li H. et al., 2020). Thus, the construction of hybrid scaffolds with fiber-reinforced structure and biomimetic ECM is of great significance in the treatment of meniscus injuries. Our team has previously demonstrated the regenerative potential of the 3D printing PCL scaffolds infused with meniscus extracellular matrix (MECM) and alginate, from which the meniscal fibrochondrocyte-loaded hybrid scaffold presented promising meniscus regeneration in the rabbit meniscectomy model (Chen et al., 2019). Given that the PCL scaffolds are not adequate to recreate an instructive microenvironment for cell growth and tissue regeneration, incorporating MECM as a bioactive material into the PCL scaffold not only can achieve a macro-/microporous hierarchical structure but also conducive to cell migration and infiltration, and may subsequently be capable enough to promote the neo-meniscal tissue formation.

In addition to biocompatibility, mechanical strength, and porous structure, the potent activity to induce stem cells to differentiate into cartilaginous cells also is essential for MTE scaffolds (Liu et al., 2019). Kartogenin (KGN), a small heterocyclic compound, is known for its prochondrogenic activity on MSCs of humans, rabbits, and rats. The introduction of KGN into the defect area may ideally induce endogenous stem/progenitor cells (ESPCs) to differentiate into cartilage cells. However, the uncontrolled delivery of bioactive factors may limit reparative potentials and cause further unwanted side effects (Yang et al., 2020). In the present study, when therapeutic drugs were intra-articularly injected into the joint, retention of these bioactive factors is relatively short and ineffective, which is mainly owing to their small molecular size (Patel et al., 2019). Therefore, the management of meniscus damages would be significantly enhanced with localized and sustained delivery of bioactive factors (Li H. et al., 2020). Liu et al. (2019) reported that injection of KGN directly into the meniscal wound area could not maintain enough concentration of this drugs for wound healing; however, a platelet-rich plasma (PRP) gel acted as a good carrier for KGN delivery and achieved augmented reparative results *in vivo*. In the study present, the development of drug delivery approaches in MTE is relatively short of examples. Therefore, there is an urgent need to develop such novel drug delivery systems (DDSs) for the sustained release of bioactive factors for meniscus regeneration. It is well known that poly(lactic-co-glycolic) acid (PLGA) microparticles are excellent DDSs to improve drug pharmacokinetics and pharmacodynamics (Martins et al., 2018). Therefore, we aim to use PLGA microparticles to improve KGN-releasing characteristics and, consequently, facilitate chondrogenesis, and achieve desired therapeutic results on meniscus defects.

The main objective of this study is to fabricate biomimetic PCL/MECM scaffolds with a controlled drug delivery system to mimic an ideal microenvironment for chondrogenesis both *in vitro* and *in vivo*. We first proposed to fabricate a composite scaffold with a fibrous hierarchical structure by utilizing 3D printing PCL scaffold as a mechanical support and MECM as a natural microenvironment for cell migration and internal infiltration. Moreover, introducing MECM components can also be expected to benefit the proliferation and cartilage matrix formation. Then, bioactive KGN was chosen to further functionalize the PCL/MECM scaffold through a PLGA microsphere (μS). The physicochemical properties of the as-prepared composite scaffolds including morphology, composition, hydrophilicity, mechanical properties, and the release profile of KGN were studied. Moreover, the biocompatibility, promigration property and chondrogenic activity of the scaffolds were also systematically evaluated. At last, we used PCL/MECM-KGN μS scaffolds to repair meniscus defects in a rabbit model, and the regenerated effects were presented and discussed. Based on such studies, it is believed that the KGN-functionalized PCL/MECM scaffolds with hierarchical structure, excellent biocompatibility, enhanced migration and chondrogenesis will be ideal substitutes for meniscus regeneration.

## Materials and Methods

### Preparation of the Scaffolds

#### Preparation of KGN μS

**FIGURE 1:**
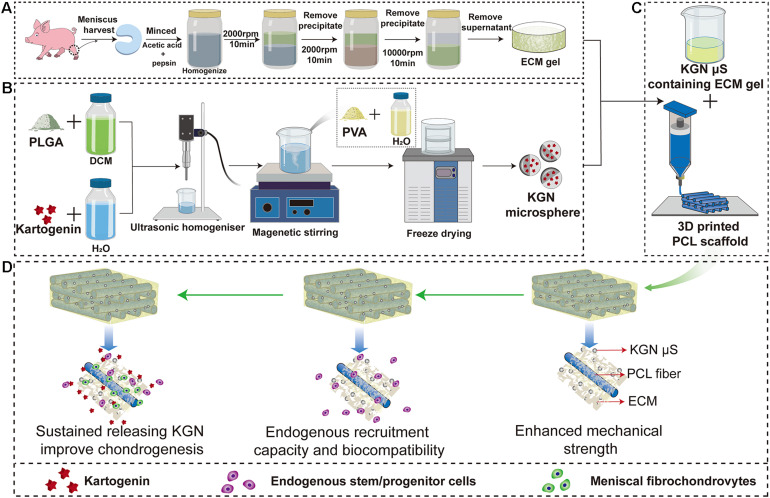
Schematic representation of the preparation process of the scaffolds. Flow chart of the preparation of the **(A)** MECM gel, **(B)** KGN-containing PLGA microspheres and **(C)** PCL/MECM-KGN μS scaffold; **(D)** Possible mechanism of meniscus regeneration. Enhanced mechanical strength, endogenous stem cells and sustained releasing KGN contributed to meniscus regeneration in these experiments. Abbreviations: MECM, meniscus extracellular matrix; KGN, kartogenin; PLGA, poly(lactic-co-glycolic) acid; μS, microspheres.

Kartogenin-loaded PLGA μS were prepared using oil-in-water (O/W) emulsion-solvent evaporation method as previously described (Wang et al., 2019; Asgari et al., 2020). In detail, 120 mg of PLGA (Daigang Biomaterial, Jinan, China) was dissolved in 2 ml of dichloromethane and then added with 2 ml mixed solution of dimethyl sulfoxide (DMSO) and dichloromethane (v/v = 1:4) containing 4 mg of KGN (Sigma-Aldrich, United States). Then, the primary emulsion was generated via an ultrasonic homogenizer (Qsonica Q125, United States) operating at an amplitude of 60% (power of 75 W) for 30 s. After this, the resultant emulsion was immediately added to 40 ml 1% (w/v) PVA solution and magnetically stirred for 12 h at room temperature to volatilize dichloromethane. After these steps, KGN μS were washed five times with distilled water and freeze dried overnight for collection (Figure 1). The KGN-free μS were fabricated following the same procedure. To determine the encapsulation efficiency (EE) of the KGN μS, the final amount of KGN in supernatants was detected by monitoring the absorbance at 287.4 nm using calculated UV spectrophotometry (Beckman, Fullerton, CA, United States) based on a pre-established KGN standard curve (Supplementary Figure 1). Then, the amount of KGN in final PLGA μS was indirectly determined by measuring the amount of KGN that remained in the PVA solution; subsequently, the amount of KGN entrapped into the microspheres was calculated according to the following formulae:

EE = $\frac{T\,o\,t\,a\,l\,a\,m\,o\,u\,n\,t\,o\,f\,K\,G\,N\,a\,d\,d\,e\,d-A\,m\,o\,u\,n\,t\,o\,f\,f\,r\,e\,e\,K\,G\,N\,a\,f\,t\,e\,r\,e\,n\,t\,r\,a\,p\,m\,e\,n\,t}{T\,o\,t\,a\,l\,a\,m\,o\,u\,n\,t\,o\,f\,K\,G\,N\,a\,d\,d\,e\,d}\times 100\%$

#### Fabrication of MECM

Decellularized MECM slurry was physicochemically prepared from swine menisci as described previously with some modifications (Yuan et al., 2016). Briefly, the menisci were harvested from a swine knee joint and washed with phosphate-buffered saline (PBS, Sigma, United States) and sterilizing with 3% hydrogen peroxide (H_2_O_2_, Sigma-Aldrich, United States). The minced menisci were treated with pepsin and acetic acid (Sigma Aldrich, United States) and homogenized at 4°C (Kinematica AG, Lucerne, Switzerland). These meniscal tissues were decellularized according to the differential centrifugation methods adopted at 2000 rpm for 10 min, 6000 rpm for 30 min, and 10,000 rpm for 30 min. These procedures were repeated five times to achieve decellularization, and the decellularized MECM slurry was stored at 4°C for later use (Figure 1).

#### Fabrication of PCL, PCL/MECM, and KGN μS Incorporated PCL/MECM Scaffolds

The pure PCL scaffolds were designed with CAD using Mimics 17.0 software to export in STL format and then fabricated using a 3D layer-by-layer fused deposition modeling (FDM) printer (FUNMAT, INTAMSYS TECHNOLOGY, China). In detail, PCL (Mw = 45,000, Sigma, United States) beads were put into the printing chamber and preheated at 90°C. The melt PCL were extruded through a heated metal nozzle (diameter, 0.25 mm) at a printing speed of 220 mm/min and finally deposited onto a receiving platform with a lay-down patten of 0°/90°/180° along the *z*-axis. Subsequently, the PCL scaffold with a fiber diameter of 250 μm and filament gap of 500 μm was produced. Then, freeze-dried KGN μS were incorporated into MECM gel with a ratio of 20 mg/ml to form KGN μS-containing MECM gels. Lastly, the PCL scaffolds were injected with liquid MECM slurry (2% w/v) with or without KGN μS and put into an ultrasonicator for another 2 h and subsequently lyophilized for collection. Finally, PCL/MECM and PCL/MECM-KGN μS scaffolds were crosslinked using carbodiimide solution [14 mM 1-ethyl-3-(3-dimethylaminopropyl) carbodiimide hydrochloride (EDAC) and 5.5 mM N-hydroxysuccinimide (NHS); Sigma] for 2 h and sterilized using ethylene oxide. All scaffolds for *in vivo* implantation were formed by crescent-shaped molds.

### Analysis of Microparticles

We incorporated goat antimouse immunoglobulin G (IgG) Alexa Fluor 594 secondary antibody into PLGA microspheres to investigate the drug distribution in the microspheres, and these microspheres were observed under a fluorescence microscope with excitation wavelengths of 565 nm. The fluorescence images of the microparticles were captured. To observe the shape and surface morphology of KGN-containing PLGA microspheres, the freeze-dried KGN μS were uniformly coated on the surface of the conductive adhesive of the sample table and observed by a scanning electron microscope [S-4800 field emission scanning electron microscope (SEM); Hitachi, Tokyo, Japan]. In order to measure the average size of the microspheres, pictures of three independent microparticle samples were captured, and ImageJ (United States) was used to calculate and present the average particle size distribution.

### Characterization of Scaffolds

#### Biochemical Analysis and Histological Staining

The prepared MECM was biochemically analyzed for total DNA, GAG, and collagen content, respectively. The DNA of MECM sample was extracted using the TIANamp Genomic DNA kit (TIANamp, Beijing, China) and then measured by the PicoGreen DNA assay kit (Invitrogen, Carlsbad, CA, United States). The content of GAG was estimated by using the Tissue GAG Total Content DMMB Colorimetry kit (Genmed Scientific Inc., Shanghai, China). The hydroxyproline assay kit (Nanjing Jiancheng, Jiangsu, China) was used to quantify collagen content. For histological assessment, 4′,6-diamidino-2-phenylindole (DAPI) and hematoxylin and eosin (H&E) staining were used to detect nucleic acids. Toluidine blue (TB) and Safranin O staining were used to determine the GAG content. The collagen distribution was identified by Sirius red staining.

#### Scanning Electron Microscopy

Scanning Electron Microscopy (S-4800 field emission scanning electron microscope; Hitachi, Tokyo, Japan) was performed to observe the morphological microstructure of KGN μS, PCL scaffolds, PCL/MECM scaffolds, and PCL/MECM-KGN μS scaffolds, respectively. Before this step, all samples were lyophilized for 24 h and then coated with Au.

#### Fourier Transform Infrared Spectroscopy

A Bruker Tensor 27 Fourier transform infrared (FTIR) spectrometer (Nicolet IS5, Germany) was used to identify the functional groups in PCL, MECM, and PCL-ECM scaffolds. Samples were cut to a size of 10 mm × 10 mm and used for conducting FTIR analysis in a reflection mode. All spectra were recorded between 4000 and 1000 cm^–1^ at a resolution of 1 cm^–1^.

#### Pore Size Distribution and Porosity Measurement

A mercury porosimeter (Micromeritics AutoPore IV 9500, United States) was used to determine pore geometry and pore volume of PCL/MECM and PCL/MECM-KGN μS scaffolds, respectively. First, the samples were completely evacuated to remove air. Then, porosimetry experiments were conducted with an equilibration time of 10 s, and mercury was forced into the pores with increasing pressure from 0.10 to 30,000 psia; after that, the pressure was reduced incrementally back to atmospheric pressure. For each group, about 0.015 g of the samples was analyzed with a 3-ml penetrometer.

#### Mechanical Testing

Scaffold samples were prepared into different sizes for mechanical testing. The 5 × 5 × 3 mm samples were used for the compression tests, which were performed using a BOSE biomechanical testing machine (BOSE 5100; TE Instruments, New Castle, DE, United States). For tensile strength, 4 × 10 2 mm specimens were measured using a uniaxial materials testing machine (Model 5969; Instron, High Wycombe, United Kingdom). The compression and tensile moduli were calculated according to the slope of the linear fit to the strain–stress curves. All tests included three parallel replicates for each group.

#### Hydrophilic Characteristic

Dynamic contact angle measurement was conducted to assess the scaffolds’ hydrophilic properties by using optical contact angle measuring and contour analysis systems (Dataphysics OCA20, Germany). Briefly, a droplet of deionized water was dropped on the sample surface, and the contact angle was captured and calculated at four time points (1, 5, 15, and 30 s) at room temperature, and five parallel sites of samples were used to analyze each group.

#### The Release of KGN From the Scaffolds

The lyophilized PCL/MECM-KGN μS scaffolds were soaked in 10 ml of PBS solution, which was shaken with 80 rpm for 28 days at 37°C. At each sampling time, 2 ml of PBS was removed by centrifugation and replaced by fresh PBS with the same amount. The supernatants were stored at −20°C until measured. The total amount of released KGN on the PCL/MECM-KGN μS scaffolds was checked by UV spectrophotometry (Beckman, Fullerton, CA, United States) at 278.4 nm based on a standard curve (Supplementary Figure 1).

### *In vitro* Cytocompatibility Studies

#### *In vitro* SMSCs Culture

Synovium-derived mesenchymal stem cells were isolated and cultured as previously described with slight modification (Moriguchi et al., 2013; Li Z. et al., 2020). In brief, the synovial tissue was obtained from the rabbit joint and thoroughly washed three times with PBS containing 1% penicillin–streptomycin (Sigma, United States) and then meticulously minced and digested with 0.25% collagenase (Gibco, United States) in Dulbecco’s modified Eagle’s medium/F12 (Corning, United States) in the incubator for 2 h at 37°C. After neutralization with Dulbecco’s modified Eagle’s medium (DMEM)/F12 with 10% fetal bovine serum (Gibco, United States), the cells were centrifuged at 1500 rpm for 10 min for collection and finally resuspended in growth medium and subsequently cultured in 25-cm^2^ flasks (Corning, United States). After 90% confluence was reached, the cells were progressed to passage after cell fusion, and passage 3 of SMSCs was used to conduct the following studies.

#### Cell Viability

The viability of SMSCs seeded onto the different scaffolds was assessed using a Live/Dead assay (Beyotime, China). Cell/scaffold composites were cultured for 4 days and then stained using a Live/Dead assay according to the manufacturer’s instructions. Briefly, 400 μl staining solution containing 2 mM calcein AM and 4 mM ethidium homodimer-1 was added to the cell/scaffold composites and incubated for 30 min at 37°C in the dark. The samples were then washed twice in PBS buffer solution for 5 min. Cells that were alive (stained green) and dead (stained red) and their distribution were observed and captured using a confocal laser scanning microscope (Leica, Germany). Cell viability was measured according to the following formula: (live cells/total cells) × 100% (*n* = 5).

#### Cell Proliferation

For cell proliferation assays, SMSCs were seeded on the scaffolds (2 × 10^4^ cells/sample) in wells of a 24-well plate and incubated for 4 h to allow for cell attachment; then, the cell/scaffold composites were transferred to another 24-well plate and cultured for 1, 3, and 7 days, respectively. In testing time point, the scaffold with cells were relocated to a new culture plate with 10% Cell Counting Kit-8 (CCK-8) work solution at 37°C and 5% CO_2_ incubator for 1 h. Subsequently, the optical density (OD) of the sample solutions (*n* = 4 per group) at 450 nm were measured by a microplate reader (Beckman, Fullerton, CA, United States).

#### Cell Adhesion and Spreading

Cell adhesion and morphology of SMSCs grown on these three groups of scaffolds were assessed by SEM and phalloidin-rhodamine and DAPI staining. P3 SMSCs (2 × 10^5^) were inoculated in different scaffolds and incubated at 37°C with 5% CO_2_ for 3 days. The cell/scaffold composites were removed and fixed with 4% paraformaldehyde for 30 min, then permeabilized with 0.3% Triton X-100 for 10 min, followed with bovine serum albumin (BSA) blockage and PBS rinse; phalloidin-rhodamine was added to stain the cytoskeletal protein F-actin overnight at 4°C and finally counterstained with DAPI for 10 min. Cell morphology and spreading were observed by using a Leica TCS-SP8 confocal microscope (Leica, Germany) to collect images. The adhesion of SMSCs cultured *in vitro* on the scaffolds were also observed by SEM. Briefly, cell/scaffold composites were fixed in 2.5% (w/v) glutaraldehyde after 3 days culture and were observed using S-4800 field emission SEM (Hitachi, Tokyo, Japan).

#### Biochemical Assays for the Secretion of GAG and Collagen

After 7, 14, and 21 days of culture, the collagen and glycosaminoglycan secretion assays were carried out. The secreted collagen and GAG were all measured both in scaffold and medium. For collagen quantification, the content of hydroxyproline was measured by the hydroxyproline assay kit (Nanjing Jiancheng Bioengineering Institute, Nanjing, China) to indicate collagen contents. For GAG quantification, the Tissue GAG Total Content DMMB Colorimetry kit (Genmed Scientific Inc., Shanghai, China) was used to detect GAG contents according to the standard curve.

### *In vitro* and *in vivo* Cell Migration

#### *In vitro* Cell Migration Assay

In order to determine the recruitment capacity of different scaffolds on SMSCs, we used a Transwell system (Corning, United States). Briefly, 100 μl of DMEM medium containing 2 × 10^4^ SMSCs was added in the upper chamber, and 600 μl of DMEM and scaffolds (10 mm × 10 mm) were placed in the lower chamber. This Transwell system was cultured for 24 h and then the migrated cells were fixed with 4% paraformaldehyde for 20 min and stained with crystal violet (2%, w/v). Six replicates of each scaffold groups were imaged. The migrated cell numbers per view were calculated by ImageJ software.

#### Endogenous MSCs Recruitment Study *in vivo*

To investigate the ability of the various scaffolds on recruiting SMSCs *in vivo*, the PCL, PCL/MECM, and PCL/MECM-KGN μS scaffolds were implanted in the meniscus of the Sprague–Dawley rats. CD73 and CD105, which were defined as MSC markers and assessed by immunofluorescence staining to determine the endogenous MSC recruitment capacity of the three scaffolds. Briefly, after the samples were harvested, we used 4% paraformaldehyde to fix them at room temperature for 30 min, and then, the target antigens were retrieved by 2% sodium citrate for 20 min and blocked by 10% goat serum. Subsequently, the samples were incubated with primary antibodies against CD73 (1:150, Novus Biologicals) and CD105 (1:150, Novus Biologicals) at 4°C overnight, followed by incubation with secondary antibodies, which were conjugated with Alexa Fluor488 and Fluor594 (1:100, Abcam, Cambridge, United Kingdom) for 1 h and DAPI (1:1000, Life Technologies) for 10 min. The samples were imaged by a TCS-SP8 confocal microscope (Leica, Germany) to determine the total cell numbers and CD29 and CD90 double-labeled cell numbers.

### Cell Chondrogenic Differentiation

#### Pellet Culture

Chondrogenic differentiation was performed according to a previously published study (Fan et al., 2008). Approximately 5 × 10^5^ SMMCs at passage 2 were centrifuged at 1500 rpm for 5 min in 15 ml Falcon tubes to form cell pellets. The pellets were maintained at 37°C with 5% CO_2_ in basal media for 24 h, following which they were put in Transwell plates placed in 24-well plates. The 24-well plates contained either PCL scaffold, PCL/MECM scaffold, or PCL/MECM/KGN-μS scaffold. Each well of the 24-well plates containing scaffold was nourished with chondrogenic differentiation medium (MSCgo^TM^, Biological Industries, Israel), and the medium was replenished every 3 days for 3 weeks. Chondrogenesis was evaluated through H&E, Alcian blue, Safranin O, and collagen II immunofluorescence staining after 21 days, respectively (*n* = 4).

#### *In vitro* Cartilage-Related Gene Expression Analysis

Cartilaginous gene expression levels of cells seeded into scaffolds after 7 and 14 days of culture were analyzed by quantitative real-time PCR (qRT-PCR). Briefly, total RNA was extracted by commercial TRIZol (Invitrogen, United States) reagent and reverse transcribed into complementary DNA (cDNA) by using the ReverTra Ace qPCR RT Kit (FSQ-201; TOYOBO) according to the manufacturer’s protocol. Then, qRT-PCR was carried out using a StepOne TM Real-Time PCR system (Applied 1113 Biosystems) with SYBR Green PCR Mater Mix (Genestar, United States). The related gene primer sequences for *Col2al*, *Col1a1*, *Sox9, Acan*, and *GAPDH* are listed in Table 1. The relative amount of the messenger RNA (mRNA) expression was normalized to housekeeping gene (GAPDH), and gene expression was calculated using the 2^–ΔΔCT^ method.

**TABLE 1:** Primer sequences used for *in vivo* chondrogenic RT-qPCR.

**Target gene**		**Sequence**
SOX9	F: 5′-3′ R: 3′-5′	GCGGAGGAAGTCGGTGAAGAAT AAGATGGCGTTGGGCGAGAT
COL 2A1	F: 5′-3′ R: 3′-5′	CACGCTCAAGTCCCTCAACA TCTATCCAGTAGTCACCGCTCT
COL 1A1	F: 5′-3′ R: 3′-5′	GCCACCTGCCAGTCTTTACA CCATCATCACCATCTCTGCCT
ACAN	F: 5′-3′ R: 3′-5′	GGAGGAGCAGGAGTTTGTCAA TGTCCATCCGACCAGCGAAA
GAPDH	F: 5′-3′ R: 3′-5′	CAAGAAGGTGGTGAAGCAGG CACTGTTGAAGTCGCAG

### *In vivo* Meniscus Repair Study

#### Animal Surgery

Animal experiments were approved by the Institutional Animal Care and Use Committee at PLA General Hospital. A total of 12 male New Zealand rabbits (8 months old, 2.5–3.0 kg) were randomly allocated into four groups: PCL group, PCL/MECM group, PCL/MECM/-KGN μS group, and positive control group (Figure 7A). The animals were first anesthetized by intramuscular injections of 160 mg ketamine and 12 mg xylazine. Major medial meniscectomy (only left with 5% of the external rim) was created and received with various groups of scaffolds implantation (Figure 7B). Those rabbits that underwent only meniscectomy were selected as positive control group. After the surgery, the animals were allowed to return to their cages and move freely. The rabbits were euthanized at 3 months and prepared for evaluations.

#### Macroscopic and Histological Evaluation

The repaired medial meniscus of the rabbit was collected for macroscopy and photographed at 3 months after implantation. Then, the regenerated menisci were fixed in 4% paraformaldehyde for 72 h. After fixation, the samples were embedded in paraffin and sectioned into 7-μm slices and stained with H&E and toluidine blue (TB), Safranin O, and Sirius Red according to standard protocols.

#### Semiquantitative Histological Scoring

To further evaluate the regenerated meniscal tissue, semiquantification of three assessment points including the reparative tissue with bonding, fibrochondrocytes existence, and Safranin O stainability were also evaluated blindly according to the Ishida score system by three independently trained researchers (Ishida et al., 2007).

### Statistical Analysis

All statistical data are presented as mean ± standard deviations, using SPSS 17.0 statistical software, using t test (two groups) or one-way analysis of variance (multiple groups) to determine statistical significance. For all analyses, *P * < 0.05 means that the difference is significant.

## Results

### Fabrication and Characterization of KGN μS

**FIGURE 2:**
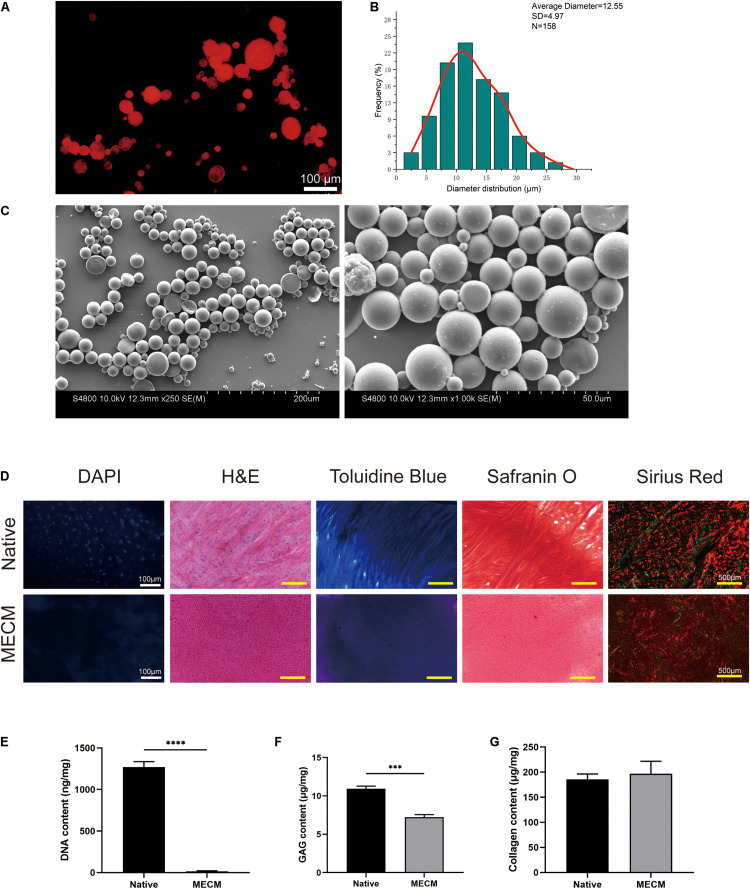
**(A)** The protein distributions in poly(lactic-co-glycolic) acid (PLGA) microspheres; **(B)** the particle size distributions of kartogenin (KGN)-incorporated PLGA microspheres; **(C)** SEM images of KGN μS; **(D)** histological staining of meniscus extracellular matrix (MECM) and native meniscus; **(E)** residual DNA, **(F)** glycosaminoglycan (GAG), and **(G)** total collagen content of MECM and native meniscus. ***p < 0.005, ****p < 0.001.

Fluorescent image (Arshady, 1991; Figure 2A) was captured, which shows successful encapsulation of goat antimouse IgG Alexa Fluor 594, and these proteins were well distributed in μS. While KGN-free μS did not exhibit obvious fluorescence in this wavelength. According to the SEM image, the μS had an average diameter of 12.55 ± 4.97 μm with a distribution ranging from 2.5 μm to roughly 30 μm (Figure 2B). The fabricated KGN μS presented a spherical morphology and also had a smooth surface (Figure 2C). A standard curve was formulated to detect KGN concentration. Analysis of KGN loading revealed that the encapsulation efficiency of KGN loading was approximately 70%.

### Characterization of MECM

The histological staining and biochemical assays presented that the decellularization process achieves complete cell removal and maintenance of collagenous and GAG component (Figures 2D–G). DAPI and H&E staining both showed complete cell nuclei absence in the MECM. The effective removal of DNA was also demonstrated by DNA quantification, which showed that the DNA component was no more than 20 ng/mg (Figures 2D,E). Positive TB and Safranin O staining results indicated that GAG contents were partly reduced in the MECM after decellularized process, which was further confirmed by the quantitative GAG assay (Figures 2D,F). Sirius red staining confirmed that collagen fibers were well preserved in the MECM, which was stained as yellow or red (Figure 2D). No significant difference of collagen contents was presented by the quantitative collagen assay (Figure 2G).

### Characterization of Hybrid Scaffolds

#### Scaffold Macro- and Microstructure

**FIGURE 3:**
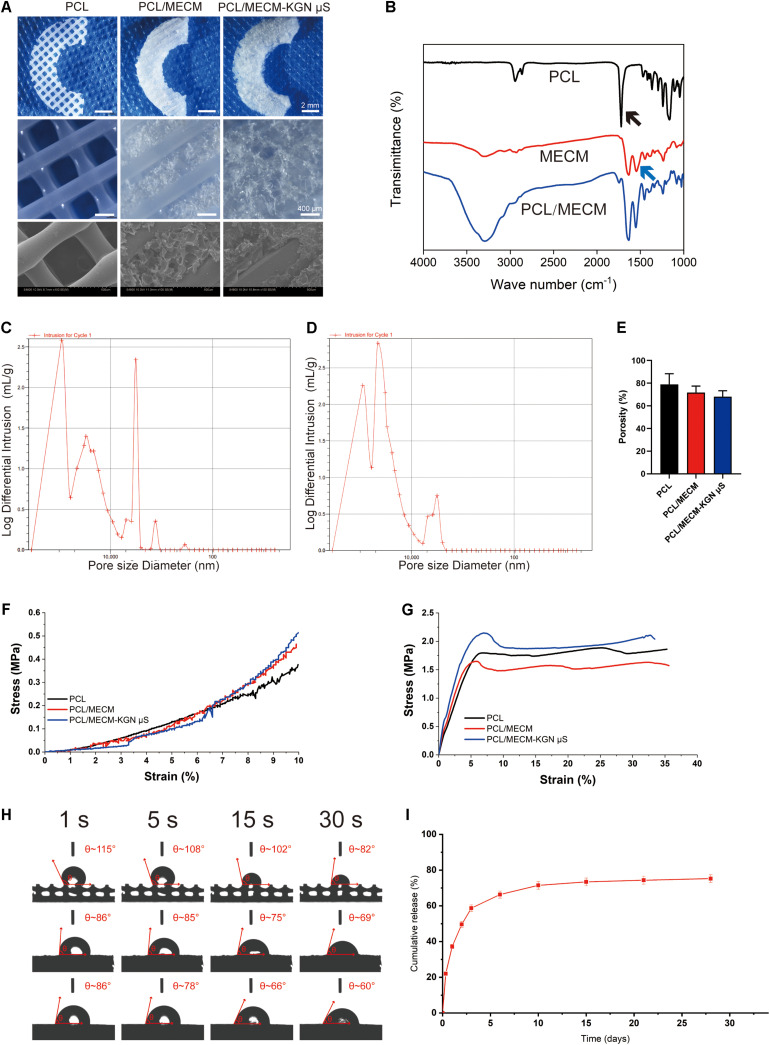
**(A)** Macroscopic and SEM images of the poly(ε-caprolactone) (PCL), PCL/meniscus extracellular matrix (MECM), and PCL/MECM-KGN μS scaffolds. **(B)** Fourier-transform infrared spectroscopy (FTIR) spectra of PCL, MECM, and PCL/MECM. Pore sizes distribution of **(C)** PCL/MECM and **(D)** PCL/MECM-KGN μS scaffolds. **(E)** Mean porosity of PCL, PCL/MECM, and PCL/MECM-KGN μS scaffolds (*n* = 3). **(F)** Compression stress–strain curve. **(G)** Tensile stress–strain curve. **(H)** Contact angles of three groups of scaffolds. Four time points (1, 5, 15, and 30 s) were chosen to vary (*n* = 3). **(I)** KGN release kinetic of PCL/MECM-KGN μS scaffold (*n* = 3).

The macro- and microstructures of the scaffolds were characterized by a stereomicroscope (SMZ2, Nikon, Japan) and SEM, as shown in Figure 3A. Crescent-shaped PCL scaffolds were orthogonally printed with square holes as shown in Figure 3A. With the introduction of MECM, the whole PCL scaffold was filled with white porous MECM structure. Microscopic structures were further observed by SEM, from which we found numerous microporous structures on gaps between PCL fibers in PCL/MECM group and PCL/MECM/-KGN μS scaffold. In order to compare the porous structure of PCL/MECM and PCL/MECM/-KGN μS samples, intrusion porosimeter was used to calculate and present pore size distribution of these two scaffolds (Figures 3C,D). The diameter of the dominant pores in the PCL/MECM/-KGN μS group was about 18 μm (Figure 3C). In comparison, the pores in PCL/MECM scaffolds had a diameter of about 32 μm (Figure 3D). These findings suggested that both PCL/MECM and PCL/MECM/-KGN μS scaffolds had hierarchical pore microstructure, and the μS-containing scaffold seems to be slightly less porous than PCL/MECM scaffold, which was consistent with SEM images. The porosity of each scaffold was also measured: 78.96 ± 7.70% for the PCL scaffold, 71.75 ± 4.64% for the PCL/MECM scaffold, and 68.17 ± 4.23% for the PCL/MECM/KGN-μS scaffold (Figure 3E).

#### FTIR

The FTIR spectra of the PCL, MECM, and PCL/MECM are shown in Figure 3B. The results showed the different characteristic peaks for PCL scaffolds, MECM scaffold, and PCL/MECM scaffolds. In the spectrogram of PCL, there was a characteristic peak of the carbonyl group (C=O) at about 1750 cm^–1^ (black arrow in Figure 3B). Characteristic peaks at 1270 and 1185 cm^–1^ corresponded to the C–O bond in PCL. As for the FTIR spectrogram of MECM, the peak at 1580 cm^–1^ (blue arrow in Figure 3B) could be recognized as a characteristic peak of the amine group (N–H bend) in MECM. Meanwhile, the peak at 1580 cm^–1^ increased, demonstrating the successful introduction of MECM into PCL scaffold.

#### Mechanical Characterization

To calculate compression and tensile moduli (Figures 3F,G), the slope of the linear region was chosen to measure the stress-strain curve. The results showed that the compression modulus of the PCL scaffold was 3.98 ± 0.34 MPa. Hybrid PCL/MECM scaffold had similar moduli (4.14 ± 0.08 MPa) to the KGN-μS-containing scaffold, whereas PCL/MECM/KGN μS scaffolds also showed a much likely modulus of 4.59 ± 0.18 MPa (Supplementary Figure 3). The tensile modulus was 20.20 ± 1.54 MPa for the PCL scaffold and 27.15 ± 1.30 MPa and 27.46 ± 2.33 MPa for the PCL/MECM and PCL/MECM/-KGN μS scaffolds, respectively (Supplementary Figure 3). As reported by Chen et al. (2019) the compression and tensile moduli of the native rabbit meniscus is 3.76 ± 0.14 MPa and 45.58 ± 1.30 MPa, respectively. Although the mechanical strength of these scaffolds was inferior to the native meniscus in terms of tensile modulus, the hybrid scaffold is desirably strong enough to withstand biomechanical forces and maintain structural integrity (Makris et al., 2011).

#### Contact Angle

The wettability of PCL, PCL/MECM, and PCL/MECM/-KGN μS scaffolds was evaluated by measuring the contact angle of distilled water. The changes in the scaffold surface hydrophobicity along with prolonged time are shown in Figure 3H and Supplementary Figure 4. The pure PCL scaffold was hydrophobic, with a contact angle of 115.61° ± 5.85°. With the introduction of MECM, the hybrid scaffold surface became more hydrophilic. The contact angles for the PCL/MECM and PCL/MECM/-KGN μS scaffolds were 86.30° ± 7.77° and 92.70° ± 6.02°, respectively. The obtained result indicated that the hydrophilicity of 3D printed PCL scaffolds was greatly enhanced by the introduction of MECM components.

#### The Release Profile of KGN

From the release curves of the scaffolds shown in Figure 3I, an initial burst release of KGN was obvious, and the majority of the drug (nearly 60%) was released within 3 days, followed by a slower, more sustained released for all drug-loaded PCL/MECM-KGN μS, with a cumulative release rate of 78% at 28 days.

### Cytocompatibility Analysis

#### Cell Viability, Proliferation, and Adhesion Assessment

**FIGURE 4:**
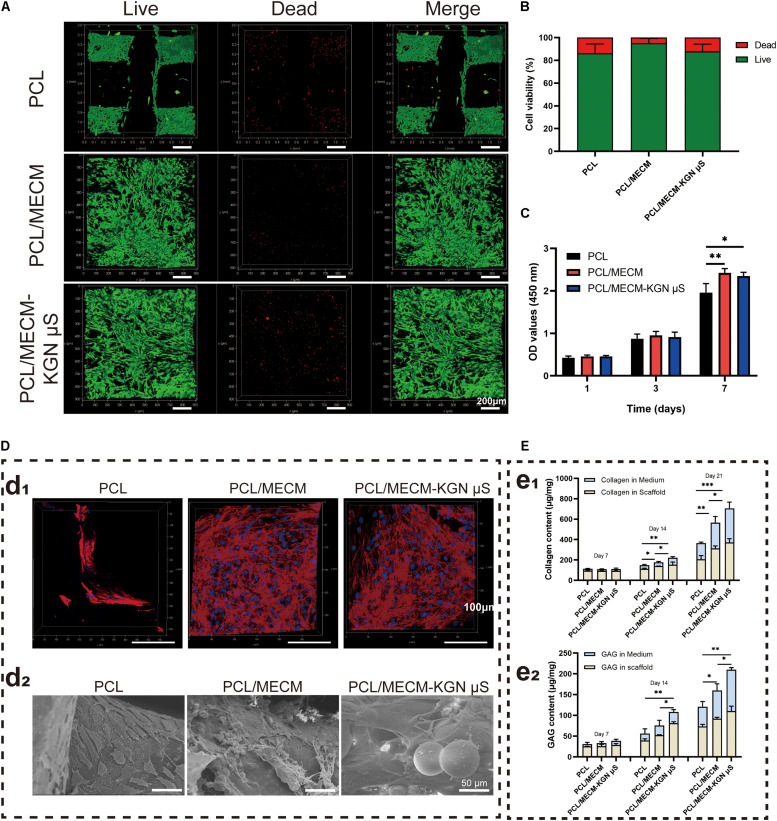
**(A)** Live/dead staining (green, live cells; red, dead cells) of synovium-derived mesenchymal stem cells (SMSCs) on the poly(ε-caprolactone) (PCL), PCL/meniscus extracellular matrix (MECM), and PCL/MECM-KGN μS scaffolds. **(B)** Cell viability analysis. **(C)** CCK-8 assay (*n* = 4) of SMSCs after 1, 3, and 7 days of culture. **(D)** Confocal morphology (red, F-actin; blue, nucleus) and SEM of the scaffolds on which SMSCs were seeded for 4 days. **(E)** Total GAG and collagen of the cell/scaffold composites at different time points (*n* = 3). *p < 0.05, **p < 0.01, ***p < 0.005.

The cytocompatibility of fabricated scaffolds was assessed by cell viability, cell proliferation, and cell adhesion assays. The live/dead staining showed that the SMSCs grew uniformly on both the PCL/MECM and PCL/MECM-KGN μS scaffolds; the MECM filling gaps between PCL fibers may provide excellent microenvironment for cell growth (Figure 4A). For PCL scaffold, the living cells were mainly arranged along the orthogonally printed fibers. A minority of the stained cells were fluorescent red (dead cells); the cell viability of various groups was also calculated by quantitative analysis, and the results exhibited that these scaffolds all have favorable viability more than 85%, indicating that all scaffolds were suitable for cell growth (Figure 4B).

Cell counting kit-8 assay was performed to quantitatively measure the proliferation of SMSCs in all types of scaffolds. As shown in Figure 4C, the OD value of cells cultured on all of the scaffolds increased with time in all the groups, and the absorbance values on the PCL/MECM and PCL/MECM/-KGN μS groups were obviously improved compared to the PCL group on day 7. These results suggested that the cell proliferation ability was greater on the MECM-containing scaffold.

A confocal laser scanning microscope was further used to evaluate the cell-spreading morphology and attachment after 72 h of seeding, and Figure 4D shows the cytoskeletal protein F-actin (red) and nucleus (blue) of cultured SMSCs on the scaffolds. Compared to the cells on the PCL scaffold, SMSCs on the MECM-containing scaffold was well distributed not only on the surfaces of PCL fibers but also in the pores of scaffolds and exhibited more fusiform morphology and pseudopods as well as a larger cell spreading area. According to SEM images of cell adhesion on the MECM-containing scaffold, the SMSCs with rough morphology were contracted together to form a stretched cell sheet (Figure 4D). These results further validated that the MECM-containing scaffolds are beneficial for cell adhesion and proliferation.

#### Biochemical Analysis for GAG and Collagen Secretion

The effects of scaffolds on the cartilaginous ECM secretion of SMSCs were evaluated by GAG and collagen quantitatively assays (Figure 4E). Evident GAG and collagen content increased along with prolonged time in each of the three scaffolds. Indeed, the secretion of the GAG and hydroxyproline was considerably higher in the PCL/MECM/-KGN μS scaffold than in the other groups after 21 days of culture in chondrogenic medium, demonstrating that the addition of KGN could strongly enhance the production of GAG and collagen, which were the main components of the meniscus matrix. Similarly, the PCL/MECM scaffold can also promote the GAG and collagen deposition to a certain extent. By day 21, total collagen and GAG in the PCL/MECM scaffold were significantly higher than in the PCL scaffold. All data indicate that the PCL/MECM and PCL/MECM/-KGN μS scaffolds are capable to stimulate the secretion of collagen and GAG by SMSCs.

### Cell Recruitment Assessment

**FIGURE 5:**
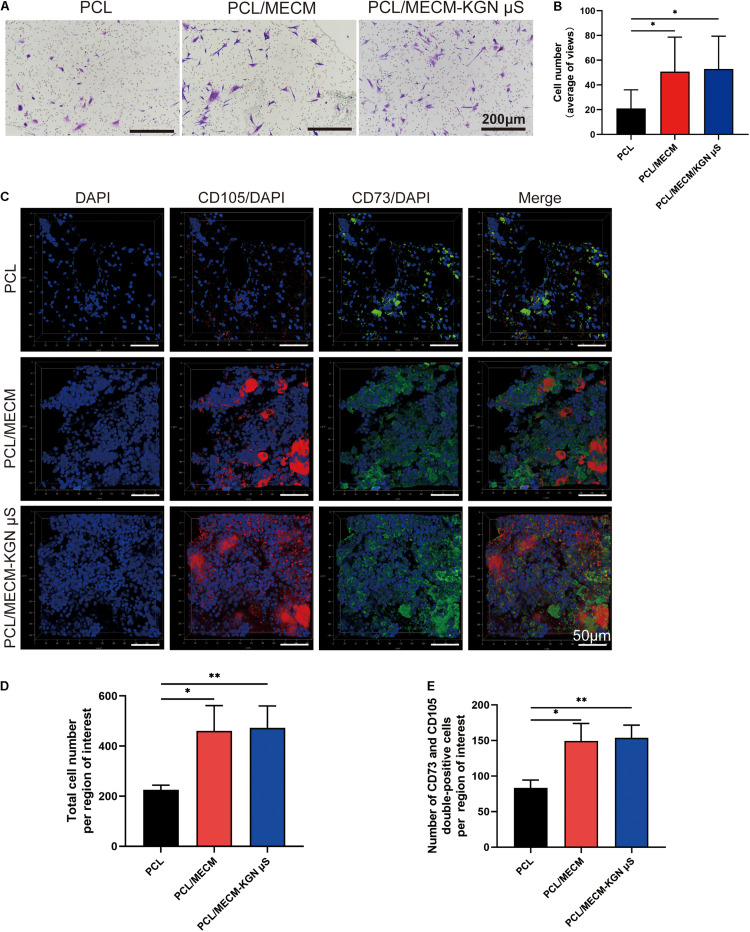
Recruitment capacity of the scaffolds on synovium-derived mesenchymal stem cells (SMSCs) *in vitro* and *in vivo*. **(A)** Effects of different scaffolds on the migration of SMSCs. The migratory cells stained with crystal violet after cultured for 24 h (*n* = 5). **(B)** Statistical analysis of average migratory cell number per region of interest from different scaffolds. **(C)** Images of CD105+/CD73+ MSCs in various implanted scaffolds 4 weeks post-surgery. **(D)** Quantification of total cell number in the injury sites. **(E)** Quantification of MSCs recruited to the injury sites. *p < 0.05, **p < 0.01.

A Transwell assay was performed to investigate stem cell migration toward different scaffolds *in vitro* (Figure 5A). Placing these three scaffolds in the lower chamber, the migrated cell numbers after 24 h were significantly higher in the PCL/MECM and PCL/MECM/-KGN μS scaffolds than that in the PCL group (Figures 5A,B). However, the recruitment capability of PCL/MECM and PCL/MECM/-KGN μS scaffolds was not significantly different. These results indicated that both the KGN-loaded scaffold and PCL/MECM scaffold could promote SMSCs migration.

The recruitment ability of scaffold *in vivo* was also verified. After 1 week of *in vivo* implantation, immunofluorescence results showed that the total cell number was higher in the PCL/MECM and PCL/MECM/-KGN μS scaffolds, with a more uniform and abundant cell distribution (Figures 5C,D). Furthermore, the more migrated ESPCs within the MECM-containing scaffold were validated by the significantly higher number of CD73 and CD105 double-positive cells (Figures 5C,E). These results collectively suggested that the MECM component can effectively enhance the capability of scaffolding system to recruit surrounding MSCs to the defect site.

### Chondrogenic Differentiation Assays

**FIGURE 6:**
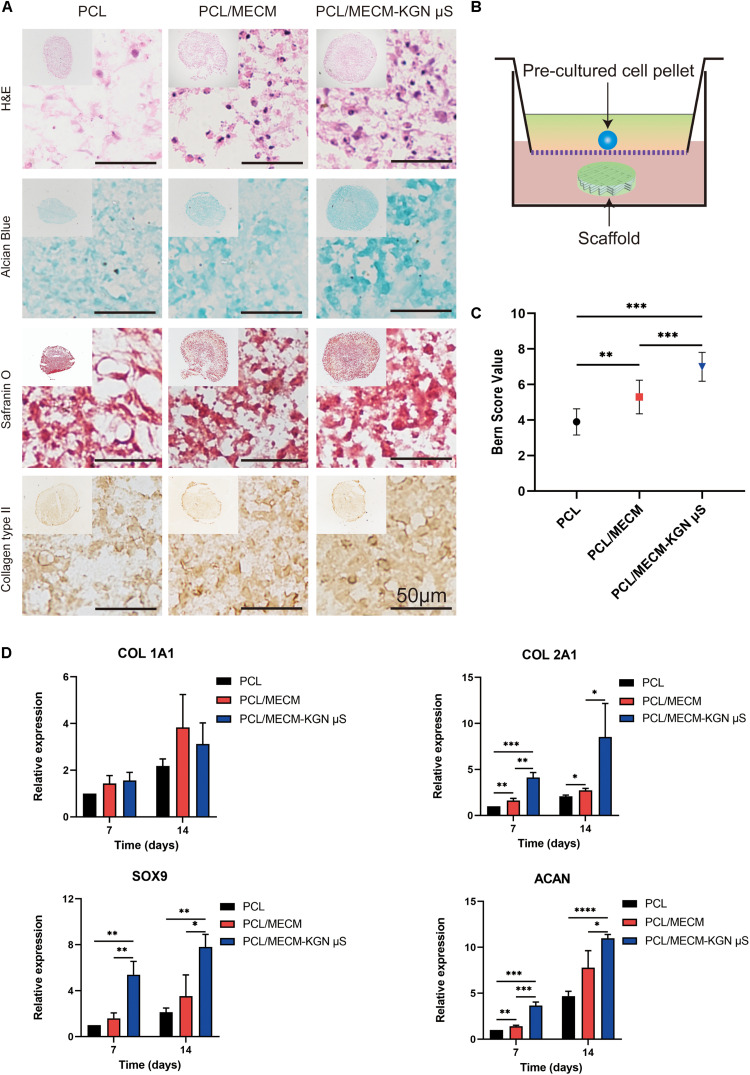
**(A)** Histological and immunohistochemical analysis of the pellets that were cocultured in **(B)** Transwell for 21 days. **(C)** Bern score for chondrogenic pellets. **(D)** Expression of Col 1A1, Col 2A1, Sox 9, and ACAN of the synovium-derived mesenchymal stem cells (SMSCs) on three different scaffolds. *p < 0.05, **p < 0.01, ***p < 0.005, ****p < 0.001.

In order to investigate the bioactivity of these scaffold, the 3D pellet system was performed, and histological and immunohistochemical staining was used to evaluate the chondrogenesis (Figure 6B). As shown in Figure 6A, H&E staining results revealed that the pellets were successfully cultured and generated. In addition, Safranin O and Alcian blue staining, which are related to proteoglycan synthesis, exhibited the predominant intensity of the pellet in the PCL/MECM-KGN μS scaffold. Furthermore, the type II collagen immunohistochemical staining results also proved that the released KGN markedly enhanced the type II collagen deposition. In terms of histological score, the Bern score evaluation showed that KGN-incorporated scaffold could prominently promote the chondrogenesis of SMSCs compared with the PCL and PCL/MECM scaffold (Figure 6C). Meanwhile, the MECM-containing scaffold also slightly enhanced the chondrogenesis of SMSCs, indicating that the instructive microenvironment was provided by MECM for SMSCs differentiation.

The expression of cartilage-associated genes of the scaffolds was also evaluated by qRT-PCR (Figure 6D). Compared with that in PCL scaffolds, the levels of *Col 2a1*, *Acan*, and *Sox9*, which are specific for cartilaginous tissues, were upregulated in the PCL/ECM groups at each time point and were also markedly enhanced after KGN incorporation in the PCL/ECM-KGN μS group. Meanwhile, the *COL1Al* expression was not significantly different within these scaffolds. Taken together, these results demonstrated that PCL/ECM-KGN μS could provide an ideal platform for chondrogenic differentiation of SMSCs.

### *In vivo* Meniscus Repair Assessment

#### Histological Staining Analysis

**FIGURE 7:**
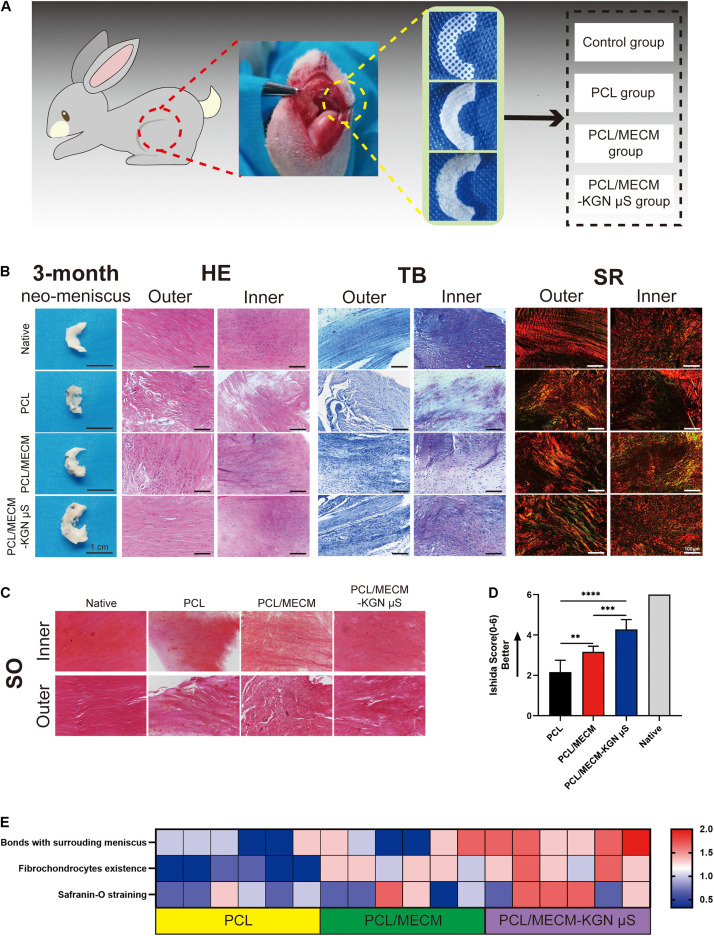
**(A)** Schematic diagram of scaffold implantation and experiment groups. **(B)** Histological staining analyses of the regenerated menisci. **(C)** Safranin O staining of the regenerated meniscus. **(D)** Ishida histological score for regenerated meniscus. **(E)** Heat map of variables of the Ishida histological scoring.

The neo-menisci were harvested 3 months after the implantation and were further evaluated by gross observation and histological staining. Compared with the PCL group, the regenerated meniscus in the PCL/MECM and PCL/ECM-KGN μS groups exhibited more complete and uniform morphology. Additionally, the meniscus regenerated in the PCL/ECM-KGN μS group was the most similar to native meniscus. The histological features of neo-menisci of different groups were also presented, respectively (Figure 7B).

In the PCL group, the results of HE staining showed only a few round-like cells and cartilage lacuna structures in the inner area of the neo-meniscal tissue, and there were basically no round-like cells in the outer area, whereas the cells were mostly long spindle-shaped cells without lacuna structure. Toluidine blue staining showed that regenerated tissues were stained weak positive. Sirius red staining also showed disordered and loosely arranged collagen fibers in both inner and outer regions. In the PCL/MECM group, the inner area of the meniscal tissue had more round-like cells and cartilage lacuna structures than the PCL group, and the outer area also exhibited a small number of round-like cells, while others are long, fusiform cells. For TB staining, newly formed meniscal tissues were stained positive. The collagen arrangement, stained with Sirius red, was improved compared to that in the PCL group. In the PCL/MECM/-KGN μS group, a large number of round-like cells can be seen in the medial area of the regenerated meniscus tissue with many cartilage lacunas structures, and the outer area is mostly long spindle-shaped cells. The TB staining in both the medial area and the outer area is strongly positive but is inferior to the native meniscus tissue. Sirius red staining shows a large number of collagen fibers with strong refractive index, and the well-organized arrangement of collagen fibers is closer to that of the normal meniscus.

#### Semiquantitative Histological Scoring

In order to further analyze the regeneration quality of the neo-menisci, we used the Ishida score to conduct a histological semiquantitative analysis. The result (Figures 7C–E) shows that the regenerated menisci in the PCL/MECM/-KGN μS group were significantly better than those in the PCL and PCL/MECM groups at the same time point.

## Discussion

The complex structure and function and limited blood supply of the native meniscus significantly hamper the successful reconstruction of this heterogeneous fibrocartilage (Li Y. et al., 2020). Tissue engineering approaches have made many advances in developing alternatives for meniscus regeneration. In a study, Liu et al. (2019) reported that KGN-loaded PRP gels successfully regenerated the wounded rabbit meniscus *in vivo* through the codelivery of stem cells and prochondrogenic factors. However, considering the drawbacks of cell-based strategies, *in situ* tissue engineering strategies that rely on leveraging the body’s innate regeneration potential provide a new avenue for injured meniscus repair and regeneration (Chen et al., 2019). Therefore, we proposed a combinatorial approach of constructing a scaffold that is capable of recruiting ESPCs, as well as maintaining chondrogenic microenvironment to regenerate the reconstructed knee meniscus. In this study, we fabricated a kartogenin-loaded hybrid scaffold by 3D printing a PCL scaffold followed by injection with the μS-incorporated MECM. We found that MECM enhanced the adhesion, proliferation, and migration of SMSCs, and the sustained release of KGN from PLGA microspheres significantly enhanced the chondrogenesis of SMSCs. The orchestrated functional microenvironment provided by this drug delivery scaffolding system in our study led to the whole meniscus regeneration in rabbits.

During the meniscus regeneration process, the implanted scaffold should provide a structural framework with suitable biophysical and biochemical properties to help the adhesion and migration of host cells. In our previous study, we fabricated and studied an ingredient and structure-mimicking hybrid scaffold derived from demineralized cancellous bone and MECM, which could act as a temporal “home” for resident cells and facilitate meniscus regeneration (Yuan et al., 2016). However, the biomechanical properties of demineralized cancellous bone are also relatively insufficient. The synthetic polymer PCL with good 3D printability, favorable mechanical support, and prolonged duration can be tuned to facilitate neo-meniscus regeneration (Shao et al., 2015). Here, the PCL fiber bundles of our scaffold presented favorable mechanical properties close to the native meniscus reported by Chen et al. (2019), as shown in Supplementary Figure 3. Furthermore, when implanted *in vivo*, the PCL fibers of the scaffold fixed at the anterior and posteriors horns can ideally resist the downward pressure and tensile strength and subsequently avoid damage and dislocation of the newly formed meniscus (Li Z. et al., 2020).

Although the MECM is not able to provide much mechanical support for meniscal tissue formation, the porous structure and various functional cues of this decellularized materials are helpful to dictate cell fate and may further help improve the meniscus regeneration (Chen et al., 2019). The microarchitectures of tissue-engineered scaffold can significantly affect the cells’ behaviors; therefore, the optimal scaffold architecture (e.g., average pore size) is a prerequisite for meniscus regeneration (Zhang et al., 2016). In our present study, MECM gel was infused into the PCL fibers and crosslinked and lyophilized to form macro–microporous structures. After the MECM infusion into the PCL scaffold, the composites had an average pore size near 30 μm and a porosity around 70%, which were believed to be helpful for cell ingrowth and proliferation (Rowland et al., 2016; Figures 3C–E). In addition, The KGN μS loaded into MECM exhibited a tendency to reduce the pore size and porosity but did not significantly change the microstructure of the whole scaffold in the present loading amount (Figures 3D,E). Another key prerequisite of the scaffold for tissue regeneration is cellular attachment and spreading onto the scaffold. The dynamic contact angle test suggested that the MECM significantly increases the hydrophilicity of composite scaffold (Figure 3H), while the loaded KGN μS did not influence the scaffold’s aqueous environment. The decreased water contact angle of the PCL/MECM scaffold can provide a more favorable microenvironment for cell adhesion. On the other hand, the biochemical cues of MECM also contribute to modulating cell behavior and providing a more instructive microenvironment. Notably, the addition of MECM supplies the constructs with excellent biocompatibility, which was further demonstrated by the results of cell viability and CCK-8 assessment shown in Figures 4A–C. *In vitro* adhesion studies also showed that the hybrid scaffold with appropriate pore structure and adhesion proteins serves as a guide for SMSCs adhesion, distribution, and proliferation (Figure 4D). Collectively, the MECM-infused PCL scaffold may retain suitable pore size, porosity, hydrophilicity, and bioactivity and further provide biomimetic microenvironment for cell infiltration, proliferation, and nutrition diffusion.

To more leveraging of innate regenerative abilities, the engineered scaffold could incorporate with bioactive factors to direct ESPCs to injury sites and aid the healing of the damaged tissue. It has been shown that the migration and infiltration of SMSCs to the injury site play a vital role in the meniscus regeneration process (Li Z. et al., 2020). In the present study, the *in vitro* and *in vivo* cell migration assay revealed that the MECM components exert recruitment capacity on SMSCs (Figures 5A–E). Although we could not figure out which kind of biomolecules in MECM are inducive to cell mobilization, the present results first proved that the MECM components can induce ESPCs migration. Some evidence showed that KGN can induce bone-marrow-derived mesenchymal stem cells (BMSCs) migration *in vitro* (Yang et al., 2019), but the results of *in vitro* and *in vivo* recruitment tests did not show that the KGN released from the PLGA microsphere exerted positive effects on cell migration, which may due to the fact that different source of stem cells or the recruitment effects of KGN was much weaker than that in MECM (Figures 5A–E).

To effectively stimulate the migrated stem cells to differentiate into matured meniscal cells, a potent chondrogenic inducer is needed. KGN has raised much attention in cartilaginous tissue engineering due to its favorable stability, cartilage protective effects, and strong chondrogenic ability (Johnson et al., 2012). However, to avoid the side effects and unwanted circulatory clearance, an ideal delivery platform that fully utilized the KGN to provide sustained chondrogenic induction is required (Zhao et al., 2020). PLGA μS were good drug carriers and capable of sustaining and localizing delivery of bioactive drugs to dictate surrounding cell behaviors (Asgari et al., 2020; Zhao et al., 2020). In the present study, the release profile of KGN-incorporated PCL/MECM hybrid scaffold exhibited an initial burst release in the early phase, followed by a slow constant release for over 28 days, which suggested that this drug delivery scaffold system can provide sustained chondrogenic environment for meniscus tissue regeneration (Figure 3I). In addition, concerning the unfavorable acidic by-product of PLGA microsphere, *in vitro* cytocompatibility assessments were also conducted. The results of live/dead assay and CCK-8 assay showed that the KGN-μS-loaded scaffold did not significantly affect the cell viability and proliferation, which proved the safety of this polymeric drug delivery system.

To validate the chondrogenic effects of the KGN-loaded composite scaffold, *in vitro* chondrogenic experiments were performed (Figure 6). After 21 days of pellet culture, we harvested and captured the pellet and histological performance and found that the PCL/MECM group promoted extracellular matrix production to some extent, but the PCL/MECM-KGN μS group exhibited more cartilaginous matrix staining than the scaffold without additional KGN (Figures 6A–C). Additionally, more chondrogenic-related gene expressions of SOX9, COL 2A1, and ACAN were also observed in the PCL/MECM-KGN μS group after 14 days of *in vitro* culture, while the PCL/MECM group can only upregulate COL 2A1 expression (Figure 6D). The cartilaginous matrix formation was also evaluated by GAG and collagen quantitative assays, and the results indicated that KGN strongly promoted ECM formation compared to the MECM only group (Figure 4E). These results further supported that KGN-loaded PCL/MECM scaffolds not only possess excellent biocompatibility but also capable of inducing chondrogenesis of stem cells. Importantly, although the optimized microstructure and hydrophilic environment of MECM are conducive to cell attachment and proliferation, a potent trophic factor that can activate and accelerate chondrogenesis of stem cells is needed and may further induce significant difference when applied *in vivo*.

In the *in vivo* studies, the better macroscopic performance of the neo-menisci in the PCL/MECM-KGN μS group is mainly owing to the superior ESPCs recruitment and differentiation ability. The poor macroscopic performance in the PCL group may be owing to the scarce biochemical cues of the PCL scaffold, which makes it hard to capture and dictate ESPCs. Compared to the PCL group, the PCL/MECM group exhibited facilitated meniscus repair to a certain extent, but the repair rate was not significantly faster, and the repaired tissue was more likely fibrous tissue. After 3 months, the regenerated meniscus of the PCL/MECM-KGN μS group were histologically more similar to the native meniscus in terms of H&E, Toluidine blue, Sirius red, and Safranin O staining. Semiquantitative histological score for the neo-menisci also showed that the PCL/MECM-KGN μS group presented better than those of the PCL and PCL/MECM groups. These findings collectively confirmed the capability of the PCL/MECM-KGN μS scaffold in facilitating meniscus regeneration. Based on our findings, we proposed a possible mechanism of meniscus regeneration induced by the present composite scaffold. First are the mobilization and migration of ESPCs to the defects. In this study, the MECM with optimized microstructure and biochemical factors successfully facilitate the enrichment of ESPCs. Second, ESPCs well proliferate and differentiate in the macro–microporous PCL/MECM, and KGN released from PLGA μS further provide potent facilitation effects on their chondrogenic differentiation. Additionally, the faster and improved cartilaginous tissue formation that was induced by KGN also further helped in achieving a positive regeneration feedback process. The accelerated meniscus-specific ECM formation may not only induce fast mechanical functions recovery but also promote the cells interaction with microenvironment and further resulting in more reparative factors releasing.

In summary, although MECM-based scaffold and PLGA microspheres have been widely used in MTE, the combination of these two components has not been seen. In our study, we first combined the KGN-loaded μS, PCL fibers, and MECM to form a hybrid scaffold, which not only provided mechanical support and excellent biocompatibility but also exerted cell recruitment ability and constantly released cartilage-inducing bioactive factors. This study also has some limitations. First, the chondroprotective effect needs to be further explored. Second, the *in vivo* results were accessed in 3 months; therefore, the scaffold should be examined in longer term similar to the natural healing process.

## Conclusion

In this study, to explore the potential of *in situ* tissue engineering strategy in meniscus regeneration, we successfully fabricated a macro–microporous PCL/MECM-KGN μS scaffold. The PCL scaffold was produced by using 3D printing technology and then injected with KGN-μS-loaded MECM to prepare a hybrid functional scaffold that aims to promote meniscus repair and regeneration. This scaffolding system with optimal microarchitecture and prochondrogenic capacity through a combination of MECM-enhancing cell migration, adhesion, and KGN facilitating chondrogenesis was evaluated both *in vitro* and *in vivo*. *In vitro* studies have demonstrated that the MECM-based scaffolds provide a favorable microenvironment to support the SMSCs migration, adhesion, and proliferation. The chondrogenic differentiation and cartilaginous matrix formation were further improved via the incorporated KGN. Moreover, *in vivo* analysis suggested that the PCL/MECM-KGN μS scaffold presented superior *in situ* meniscus regeneration. To conclude, the PCL/MECM-KGN μS scaffolding system may be a promising small molecule-based alternative, which hold great promise for MTE in the future.

## Data Availability Statement

The original contributions presented in the study are included in the article/Supplementary Material, further inquiries can be directed to the corresponding author/s.

## Ethics Statement

The animal study was reviewed and approved by the Institutional Animal Care and Use Committee at PLA General Hospital.

## Author Contributions

HL and QG did the conceptualization. HL, ZL, and ZYa did the methodology. HL, ZL, ZYu, and CG did the investigation. ZYa, LF, PL, and ZYu did the resources. HL wrote. SL and QG edited. All authors contributed to the article and approved the submitted version.

## Conflict of Interest

The authors declare that the research was conducted in the absence of any commercial or financial relationships that could be construed as a potential conflict of interest.
