# Supplementary material for: 3D Printed Poly(ε-Caprolactone)/Meniscus Extracellular Matrix Composite Scaffold Functionalized With Kartogenin-Releasing PLGA Microspheres for Meniscus Tissue Engineering
Source: Front Bioeng Biotechnol. 2021 Apr 30;9:662381. doi: 10.3389/fbioe.2021.662381 (PMC8119888; doi:10.3389/fbioe.2021.662381)
Supplement: Supplementary file 1 [file Table_1.docx]

**3D printed poly(ε-caprolactone)/ meniscus extracellular matrix composite scaffold functionalized with kartogenin-releasing PLGA microspheres for meniscus tissue engineering**

Hao Li^1,2†^, Zhiyao Liao^1,2†^, Zhen Yang^1,2^, Cangjian Gao^1,2^, Liwei Fu^1,2^, Pinxue Li^1,2^, Tianyuan Zhao^1,2^, Fuyang Cao^1,3^, Wei Chen^1,2^, Zhiguo Yuan^4^, Xiang Sui^1^, Shuyun Liu^1^, Quanyi Guo^1,2,*^

1: Institute of Orthopedics, the First Medical Center, Chinese PLA General Hospital; Beijing Key Lab of Regenerative Medicine in Orthopedics; Key Laboratory of Musculoskel etal Trauma & War Injuries PLA; No. 28 Fuxing Road, Haidian District, Beijing 100853, China

2: School of Medicine, Nankai University, Tianjin 300071, China

3: Department of Orthopedics, the First Affiliated Hospital of Zhengzhou University, 1 Jian East Road, Eqi District, Zhengzhou 450052, China

4: Department of Bone and Joint Surgery, Renji Hospital, School of Medicine, Shanghai Jiaotong University, Shanghai, China

*: Corresponding authors: Quanyi Guo, Institute of Orthopedics, The First Medical Center, Chinese PLA General Hospital, Beijing Key Lab of Regenerative Medicine in Orthopedics, Key Laboratory of Musculoskeletal Trauma and War Injuries PLA, No. 28 Fuxing Road, Haidian District, Beijing 100853, China. School of Medicine, Nankai University, Tianjin 300071, China. Email address: doctorguo_301@163.com.

†: These authors contributed equally.

**Materials and Methods**

**1. Assessment of KGN standard curve.**

The KGN dissolved in dimethyl sulfoxide (DMSO) and dichloromethane (v/v=1:4) was gradient diluted from 2.5 μmol/L to 0.001220703 μmol/L and each solution were detected by monitoring the absorbance at 287.4 nm using calculated a UV spectrophotometry (Beckman, Fullerton, CA). The standard curve was formulated by Origin 2018 statistical software.

**2. Characterization of PLGA microspheres**

SEM (S-4800 field emission scanning electron microscope; Hitachi, Tokyo, Japan) was performed to observe the morphological microstructure of PLGA microspheres. The PLGA microspheres were observed under fluorescence microscope with excitation wavelengths of 565 nm and were also observed by optical microscope.

**3. Mechanical testing**

Scaffold samples were prepared into different sizes for mechanical testing. The 5×5×3mm samples were used for the compression tests, which were performed using a BOSE biomechanical testing machine (BOSE 5100; TE Instruments, New Castle, DE, USA). For tensile strength, 4×10 2 mm specimens were measured using a uniaxial materials testing machine (Model 5969; Instron, High Wycombe, UK). The compression and tensile modulus were calculated according to the slope of the liner fit to the strain-stress curves. All tests included three parallel replicates for each group.

**4. Hydrophilic characteristic**

Dynamic contact angle measurement was conducted to assess the scaffolds’ hydrophilic properties by using Optical contact angle measuring and contour analysis systems (Dataphysics OCA20, Germany). Briefly, a droplet of deionized water was dropped on sample surface and the contact angle was captured and calculated at four time points (1s, 5s, 15s and 30s) at room temperature, and five parallel sites of samples were used to analyze each group.

**Results**

**1. KGN standard curve**

Based on the data of absorbance of gradient diluted KGN solutions, the standard curve was calculated and presented in Figure S1.

**2. Characterization of PLGA microspheres**

According to the SEM and optical microscope image, the fabricated PLGA μS presented a spherical morphology and also had a smooth surface (Figure S2A, B, D). Fluorescent image (Figure S2C) was captured which show very few fluorescence, which indicated that PLGA μS do not present fluorescence with excitation wavelengths of 565 nm.

**3. Mechanical testing**

To calculate compressive and tensile moduli (Figure 3F, G), the slope of the liner region was chosen to measure of the stress−strain curve. The results showed that the compressive modulus of PCL scaffold was 3.98±0.34 MPa. Hybrid PCL/MECM scaffold had similar moduli (4.14±0.08 MPa) to KGN μS containing scaffold, whereas PCL/MECM/KGN-μS scaffolds also showed a much likely modulus of 4.59±0.18 MPa (Figure S3). For tensile modulus, the tensile modulus was 20.20±1.54 MPa for the PCL scaffold, and 27.15±1.30 MPa and 27.46±2.33 MPa for PCL/MECM, PCL/MECM/-KGN μS scaffold, respectively (Figure S3).

**4. Hydrophilic characteristic**

The wettability of PCL, PCL/MECM, PCL/MECM/-KGN μS scaffold were evaluated by measuring the contact angle of distilled water. And the change in the scaffold surface hydrophobicity along with time prolonged are shown in Figure 3H and S4. The pure PCL scaffold was hydrophobic, with a contact angle of 115.61° ± 5.85°. With the introduction of MECM, the hybrid scaffold surface became more hydrophilic. The contact angles for the PCL/MECM, PCL/MECM/-KGN μS scaffold were 86.30°±7.77° and 92.70°±6.02°, respectively. The obtained result indicated that the hydrophilicity of 3D printed PCL scaffolds was greatly enhanced by the introduction of MECM components.





**Figure S1.** KGN standard curve. (n=3)


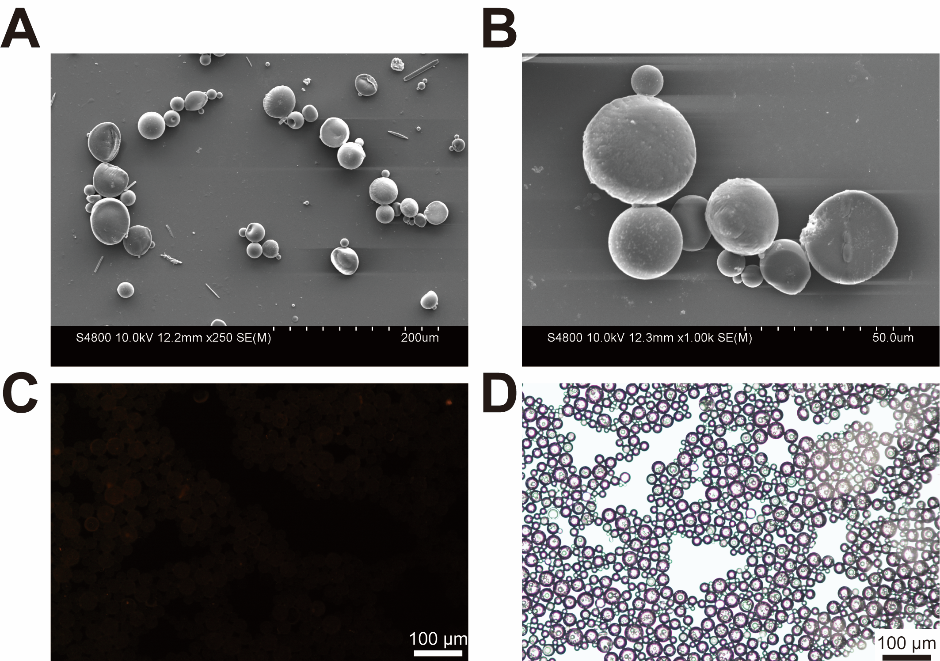


**Figure S2.** Characterization of PLGA microspheres. (A) and (B) SEM images of PLGA μS; (C)The fluorescent image; (D) The optical microscope image.


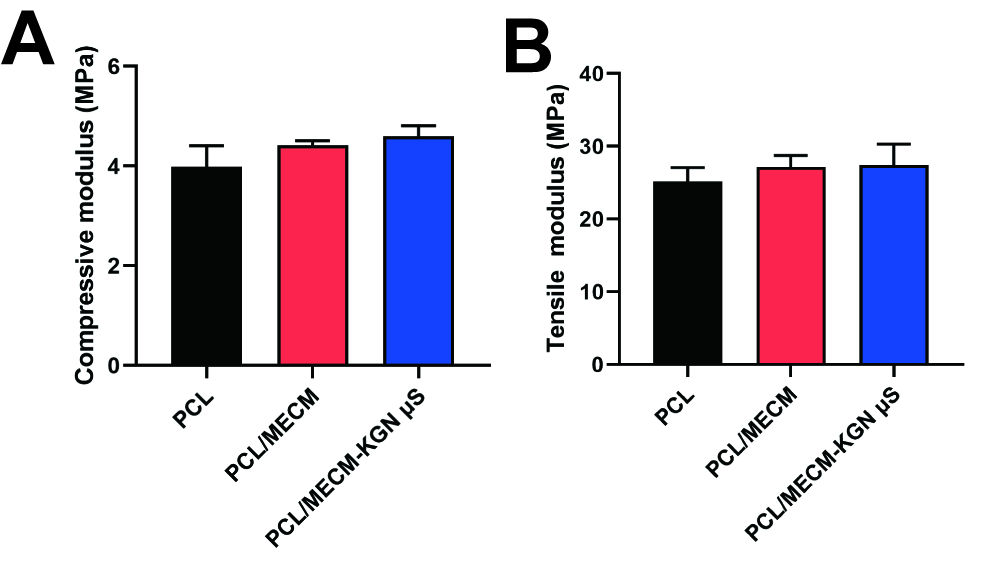


**Figure S3.** Compression (A) and tensile moduli (B) of the scaffolds (n=3).


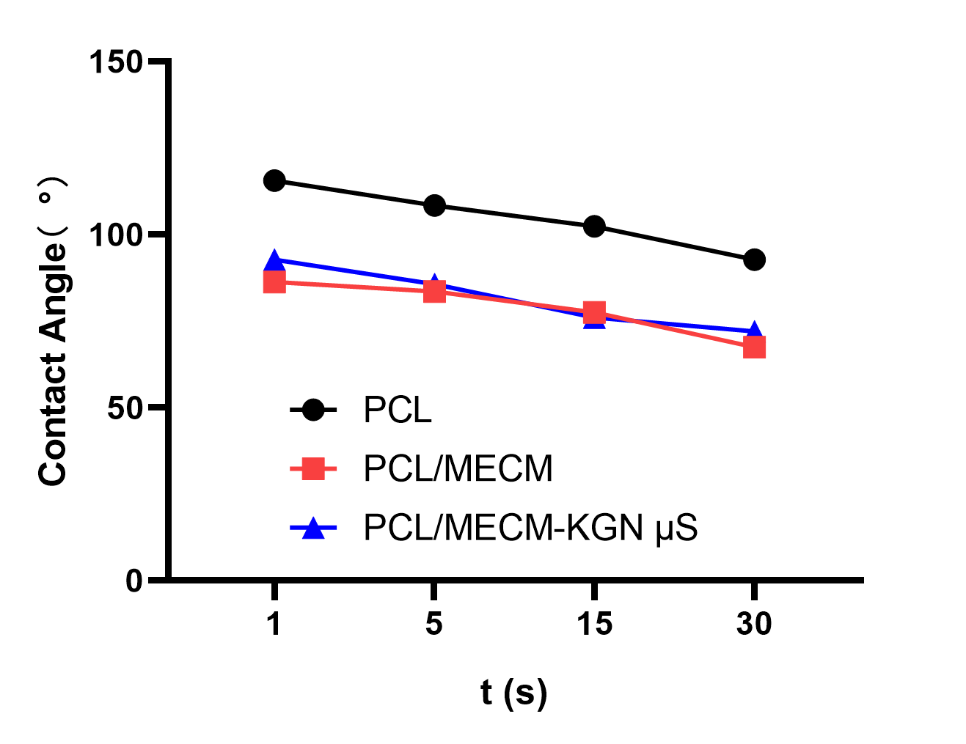


**Figure S4.** Contact angles of three groups of scaffolds. Four time points (1s, 5s, 15s, 30s) were chosen to vary (n=3);
